# Surgical resection versus transarterial chemoembolization for patients with hepatocellular carcinoma beyond Milan criteria: prognostic role of tumor burden score

**DOI:** 10.1038/s41598-023-41068-7

**Published:** 2023-08-24

**Authors:** Shu-Yein Ho, Po-Hong Liu, Chia-Yang Hsu, Yi-Hsiang Huang, Hao-Jan Lei, Jia-I Liao, Chien-Wei Su, Ming-Chih Hou, Teh-Ia Huo

**Affiliations:** 1 Division of Gastroenterology and Hepatology, Min-Sheng General Hospital, Taoyuan, Taiwan; 2 Department of Medical Research, Taipei Veterans General Hospital, No. 201, Sec. 2, Shipai Rd, Taipei, 11217 Taiwan; 3 School of Medicine, National Yang Ming Chiao Tung University, Taipei, Taiwan; 4 Department of Internal Medicine, University of Texas Southwestern Medical Center, Dallas, TX USA; 5 Department of Medicine, Renown Medical Center, Reno, NV USA; 6 Division of Gastroenterology and Hepatology, Department of Medicine, Taipei Veterans General Hospital, Taipei, Taiwan; 7 Institute of Clinical Medicine, National Yang Ming Chiao Tung University, Taipei, Taiwan; 8 Department of Surgery, Taipei Veterans General Hospital, Taipei, Taiwan; 9 Institute of Pharmacology, National Yang Ming Chiao Tung University, Taipei, Taiwan

**Keywords:** Liver cancer, Cancer therapy

## Abstract

Tumor burden score (TBS) has been recently introduced to indicate the extent of tumor burden in different cancers, but its role in advanced hepatocellular carcinoma (HCC) is unclear. We aimed to determine the prognostic role of TBS in patients with HCC beyond the Milan criteria receiving surgical resection (SR) or transarterial chemoembolization (TACE). A total of 1303 newly diagnosed HCC patients beyond Milan criteria receiving SR or TACE as the primary therapy were retrospectively analyzed. Independent prognostic predictors were examined by the multivariate Cox proportional hazards model. SR was associated with better overall survival compared with TACE in these patients. Multivariate Cox analysis of the entire cohort revealed that age > 66 years (hazard ratio [HR]: 1.145, 95% confidence interval [CI]: 1.004–1.305, *p* = 0.043), serum α-fetoprotein > 200 ng/mL (HR: 1.602, 95% CI: 1.402–1.831, *p* < 0.001), performance status 2–4 (HR: 1.316, 95% CI: 1.115–1.553, *p* < 0.001), medium TBS (HR: 1.225, 95% CI:1.045–1.436, *p* = 0.012), high TBS (HR: 1.976, 95% CI: 1.637–2.384, *p* < 0.001), albumin-bilirubin (ALBI) grade 2–3 (HR: 1.529, 95% CI: 1.342–1.743, *p* < 0.001), presence of vascular invasion (HR: 1.568, 95% CI: 1.354–1.816, *p* < 0.001), and TACE (HR: 2.396, 95% CI: 2.082–2.759, *p* < 0.001) were linked with decreased survival. SR consistently predicted a significantly better survival in different TBS subgroups. TBS is a feasible and independent prognostic predictor in HCC beyond the Milan criteria. SR provides better long-term outcome compared with TACE in these patients independent of TBS grade, and should be considered as the primary treatment modality in this special patient group.

## Introduction

Hepatocellular carcinoma (HCC) remains one of the difficult-to-treat cancers with a rising global incidence in 2020^1^. The prognosis of HCC is usually poor because the majority of patients are diagnosed at an advanced cancer stage. Chronic hepatitis B and C virus (HBV, HCV) infection, alcoholism and non-alcoholic fatty liver disease are the main etiologies of HCC^2^. Of the recommended treatments, surgical resection (SR), liver transplantation and local ablation therapy are indicated for early-stage HCC. For patients with intermediate or advanced stage, transarterial chemoembolization (TACE), targeted therapy and immunotherapy are usually suggested^3,4^.

According to current HCC practice guidelines, TACE is the primary treatment for intermediate stage HCC beyond the Milan criteria (tumor less than 5 cm in diameter or 3 or fewer tumors less than 3 cm in diameter)^5,6^. Notably, SR is typically indicated for patients with resectable HCC, but its role in patients beyond the Milan criteria remains controversial. Recent studies showed that SR is a safe and effective treatment in patients with large tumor burden and good liver functional reserve, and may provide a better outcome in selected HCC patients beyond the Milan criteria^7–11^.

The extent of tumor burden is an important concern in treatment selection for HCC. Traditionally, tumor burden is depicted as the concurrent diameter of tumor and number of nodules in most HCC staging systems. Several parameters, such as up-to-seven criteria, up-to-eleven criteria and seven-eleven criteria, were used to assess the extent of tumor involvement in HCC^12–14^. However, these parameters are categorical in fashion which may thus be inferior in prognostic prediction compared with those of continuous scores. Investigators have proposed to use a wider continuum of tumor size and number variable as a prognostic tool to assess tumor burden and to further improve prognostic stratification. Recently, Sasaki et al. proposed the metro-ticket model which utilized a continuous variable of tumor burden score (TBS) to discriminate survival in colorectal cancer patients with liver metastasis^15^. Although TBS has been evaluated in different clinical settings of HCC^16–19^, patients beyond the Milan criteria are a highly heterogeneous disease group with a broad range of tumor burden, and their clinical outcomes are quite diverse^20^. Up to date, very few studies compared the differences between SR and TACE in HCC patients beyond the Milan criteria in relation to TBS. In this study, we aimed to investigate the prognostic role of TBS in patients beyond the Milan criteria undergoing SR or TACE.

## Methods

### Patients

A total of 1,303 newly diagnosed HCC patients beyond the Milan criteria from 2002 to 2019 receiving SR or TACE as the primary anti-cancer therapy at Taipei Veterans General Hospital were prospectively enrolled and retrospectively analyzed. Of these patients, 550 received SR and 753 received TACE treatment. Their baseline information including age, gender, serum biochemistry, etiology of liver disease, tumor burden (tumor number and diameter, tumor burden score), serum α-fetoprotein (AFP) level, vascular invasion, liver functional reserve, performance status, and cancer stages, were recorded at the time of diagnosis. All patients in the SR group had a confirmed diagnosis by pathological examination. Patient survival was evaluated every 3 months until death or drop out from the follow-up program. This study has been approved by the institutional review board of Taipei Veterans General Hospital, Taiwan, and complies with the standards of Declaration of Helsinki and current ethical guidelines. The waiver of informed consent was approved by the institutional review board of Taipei Veterans General Hospital; patient’s personal information was anonymized and de-identified prior to the analysis.

### Definition

HCC was diagnosed by typical radiological findings such as arterial hyper-enhancement in arterial phase and delayed wash-out in venous phase from dynamic computed tomography (CT) or magnetic resonance imaging (MRI), or by histological confirmation if imaging features were atypical^5,21^. HBV-related HCC was defined as positive serum HBsAg and negative anti-HCV and alcoholism. Patients who were seropositive for anti-HCV and seronegative for HBsAg were denoted as HCV-related HCC. The Child-Turcotte-Pugh (CTP) classification and albumin-bilirubin (ALBI) score were used to assess the severity of liver dysfunction. The performance status scale developed by the Eastern Cooperative Oncology Group was recorded from 0 (fully active, able to carry on all daily performance without restriction) to 5 (dead)^22^.

TBS was denoted as the distance from the origin on a Cartesian plane that incorporated maximum tumor size and number of liver lesions. The calculation of TBS was as follows: TBS^2^ = [Maximum tumor diameter]^2^ + [number of liver lesions]^2^^15,17^. The cutoff value of low/medium TBS and medium/high TBS were 5.83 and 11.81, respectively. ALBI score was calculated according to the following equation = 0.66 × log_10_bilirubin (μmol/L) − 0.085 × albumin (g/L). The cutoffs of ALBI grade 1/2 and grade 2/3 were − 2.60 and − 1.39, respectively^23–25^. Vascular invasion was defined by the presence of thrombus adjacent to the tumor in the portal vein or with blurring boundary confirmed by at least one imaging modality^26^.

### Treatments

The selection criteria for SR required tumor nodule (1) confined to single lobe, or tumors involving no more than 3 Healy’s segment, (3) no main portal vein thrombosis or distant metastasis, (4) CTP class A or B, with < 20% retention rate of indocyanine green at 15 min after injection. The inclusion criteria for TACE were (1) patients who were unsuitable or refused surgery, (2) no main portal vein thrombosis or distant metastasis, and (3) CTP class A or B. The Seldinger’s technique of arterial embolization was administered as the standard TACE procedure described in previous studies^9,10,18^. After tumor stain was identified, infusion of a mixture of 20–30 mg adriamycin (Carlo Erba, Milan, Italy) and 5–10 mL lipiodol (Laboratoire Guerbet, Villepinte, France) was performed after the artery supplying the tumor was catheterized superselectively. Sufficient amount of emulsion and 2- to 3-mm strips of Gelfoam (Upjohn, Kalamazoo, MI, USA) were delivered to the tumor area until complete flow stagnation was achieved. After SR or TACE, post-treatment follow-up imaging including liver sonography, dynamic CT or MRI, and serum AFP level, were performed every 3–4 months or more frequently if necessary.

### Statistics

Continuous variables were evaluated by the Mann–Whitney rank sum test and were expressed as mean ± SD. The Fisher’s exact test or Chi-squared test was used to assess the comparison of categorical variables. The survival of different patient groups was determined by the Kaplan–Meier method with log-rank test. Independent prognostic predictors associated with survival were analyzed by the multivariate Cox proportional hazards model to determine the adjusted hazard ration (HR) and 95% confidence interval (CI). IBM SPSS Statistics for Windows, version 21.0 (IBM Corp., Armonk, NY, USA), was used for statistical analysis. A *p* value < 0.05 was considered statistically significant.

## Results

### Patient characteristics

**Figure 1:**
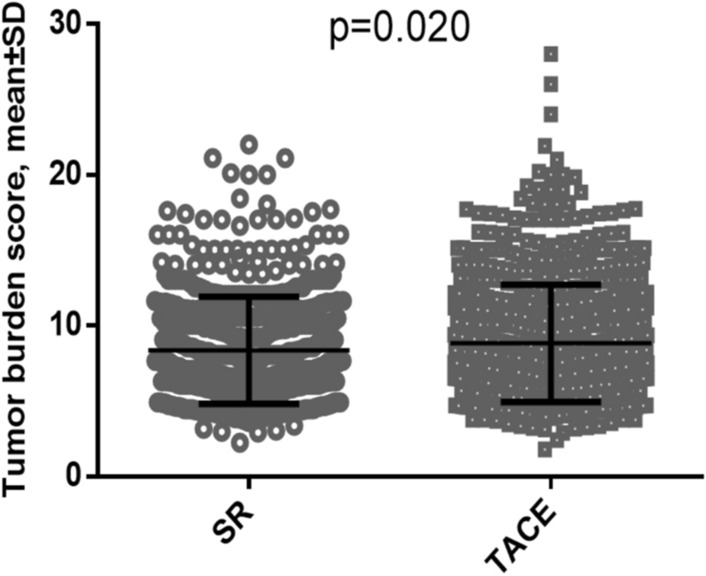
Distribution of tumor burden score (TBS) in hepatocellular carcinoma patients beyond the Milan criteria undergoing surgical resection (SR) and trans-arterial chemoembolization (TACE). The SR group had smaller mean tumor burden score compared with the TACE group (*p* = 0.020).

Table 1 shows the comparison of baseline characteristics of patients undergoing SR or TACE. Patients in the SR group were younger (*p* < 0.001), more often had HBV infection (*p* < 0.001), good performance status (*p* < 0.001), larger tumor diameter (*p* < 0.001), higher albumin level (*p* < 0.001), higher platelet count (*p* < 0.001), better liver functional reserve (CTP class A and ALBI grade 1; both *p* < 0.001), and lower bilirubin level (*p* < 0.001) than the TACE group. Alternatively, patients undergoing TACE more often had multiple tumors (*p* < 0.001) and vascular invasion (*p* < 0.001) compared with the SR group. There was also significant difference in the distribution of cancer stage between the two groups (*p* < 0.001). In addition, SR group had lower mean TBS compared with the TACE group (Fig. 1; *p* = 0.02).

**Table 1:** Comparison of baseline characteristics of patients with HCC beyond Milan criteria undergoing surgical resection (SR) or transarterial chemoembolization (TACE) (n = 1303).

Variables	SR (n = 550)	TACE (n = 753)	*p* value
Age (years, mean ± SD)	61 ± 13	67 ± 13	< 0.001
Male/female, n (%)	429/121 (78/22)	600/153 (80/20)	0.505
Etiologies of liver disease, n (%)			0.065
HBV	317(58)	340 (45)	< 0.001
HCV	79 (14)	187 (25)	
HBV + HCV	18 (3)	32 (4)	
Others	136 (25)	194 (16)	
Performance status (0/1/2/3/4), n (%)	380/128/33/9/0 (58/14/3/25/0)	414/170/125/36/8 (55/22/17/5/1)	< 0.001
Multiple nodules, n (%)	184 (34)	421 (56)	< 0.001
Tumor diameter > 5 cm, n (%)	450 (82)	115 (34)	< 0.001
Tumor diameter, mean ± SD	8.10 ± 3.7	8.07 ± 4.3	0.913
Tumor burden score (TBS)			0.297
Low	135 (25)	184 (24)	
Medium	300 (55)	385 (52)	
High	115 (20)	184 (24)	
Tumor burden score, mean ± SD	8.34 ± 3.53	8.83 ± 3.89	0.020
Serum AFP (ng/mL), mean ± SD	19720 ± 164380	28074 ± 173898	0.381
Serum AFP > 400 ng/mL, n (%)	189 (34)	259 (34)	0.990
Vascular invasion, n (%)	107 (20)	198 (26)	0.004
Laboratory values, mean ± *SD*			
Alanine transaminase (U/L)	68 ± 117	70 ± 73	0.731
Albumin (g/dL)	3.9 ± 0.5	3.6 ± 0.6	< 0.001
Total bilirubin (mg/dL)	0.86 ± 0.79	1.11 ± 1.10	< 0.001
Platelets (1,000/μL)	211 ± 90	184 ± 100	< 0.001
INR of prothrombin time	1.08 ± 0.58	1.08 ± 0.13	0.935
Creatinine (mg/dL)	1.02 ± 0.54	1.16 ± 0.95	0.001
CTP class (A/B/C), n (%)	502/48/0 (91/9/0)	576/159/18 (76/21/3)	< 0.001
ALBI grade (1/2/3), n (%)	314/228/8 (56/42/2)	249/461/43 (33/61/6)	< 0.001
BCLC stage (B/C/D), n (%)	355/236/9 (56/42/2)	288/414/51 (38/55/7)	< 0.001

*ALBI* albumin-bilirubin, *AFP* alpha-fetoprotein, *CTP* Child-Turcotte-Pugh, *HBV* hepatitis B virus, *HCV* hepatitis C virus, *INR* international normalized ratio, *SD* standard deviation.

### Survival analysis

**Figure 2:**
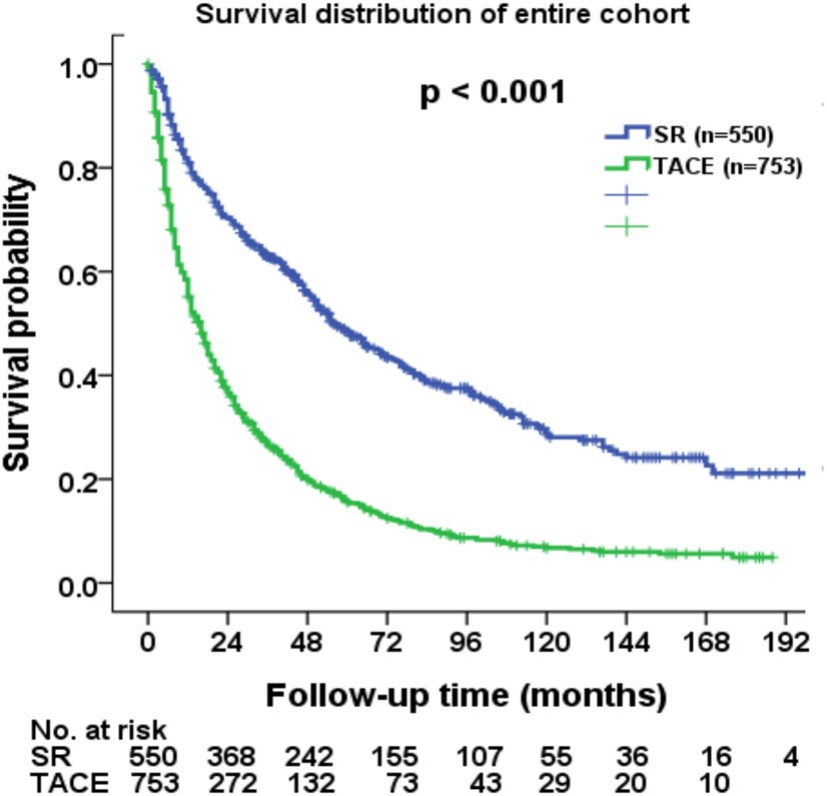
Comparison of overall survival in HCC patients beyond the Milan criteria undergoing surgical resection (SR) and transarterial chemoembolization (TACE). SR group had a better survival compared with TACE group (*p* < 0.001).

The mean and median follow-up period were 55 and 26 months, respectively. A total of 994 (76%) patients died at the time of analysis. The median survival were 56 months in SR group and 16 months in TACE group. Comparison of survival distribution between SR and TACE group is shown in Fig. 2. Patients undergoing SR had better long-term survival compared with those undergoing TACE (*p* < 0.001). The 1-, 3- and 5-year overall survival were 82%, 63%, and 49% in SR group, and 59%, 27%, and 16% in TACE group, respectively.

**Figure 3:**
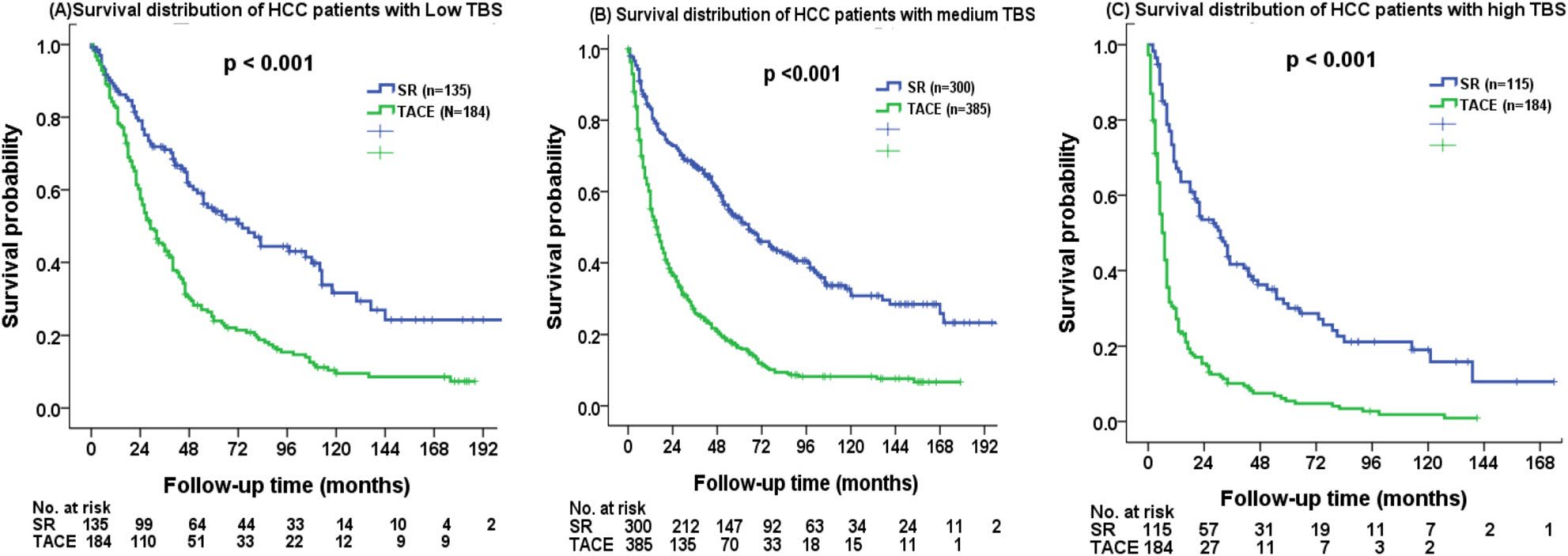
Comparison of overall survival in HCC patients beyond the Milan criteria undergoing surgical resection (SR) and transarterial chemoembolization (TACE) based on (**A**) low TBS, (**B**) medium TBS, and (**C**) high TBS. SR group consistently had a better survival compared with TACE group in patients with different TBS distributions (all *p* < 0.001).

The analysis was further stratified by TBS. In subgroup analysis of patients with low TBS, SR group had lower risk of mortality compared with TACE group (Fig. 3A, p < 0.001). The 1-, 3-, 5-year overall survival were 89%, 72%, and 55% in SR group, and 83%, 45% and 25% in TACE group, respectively. Of the patients with medium TBS, SR group had better overall survival compared with those undergoing TACE (Fig. 3B, p < 0.001). The 1-, 3-, 5-year overall survival were 84%, 67%, and 53% in SR group, and 60%, 27%, and 16% in TACE group, respectively. The SR group consistently had better long-term survival compared with TACE group among patients with high TBS (Fig. 3C, p < 0.001). The 1-, 3-, 5-year survival were 69%, 42%, and 32% in SR group, and 30%, 10% and 6% in TACE group, respectively.

**Figure 4:**
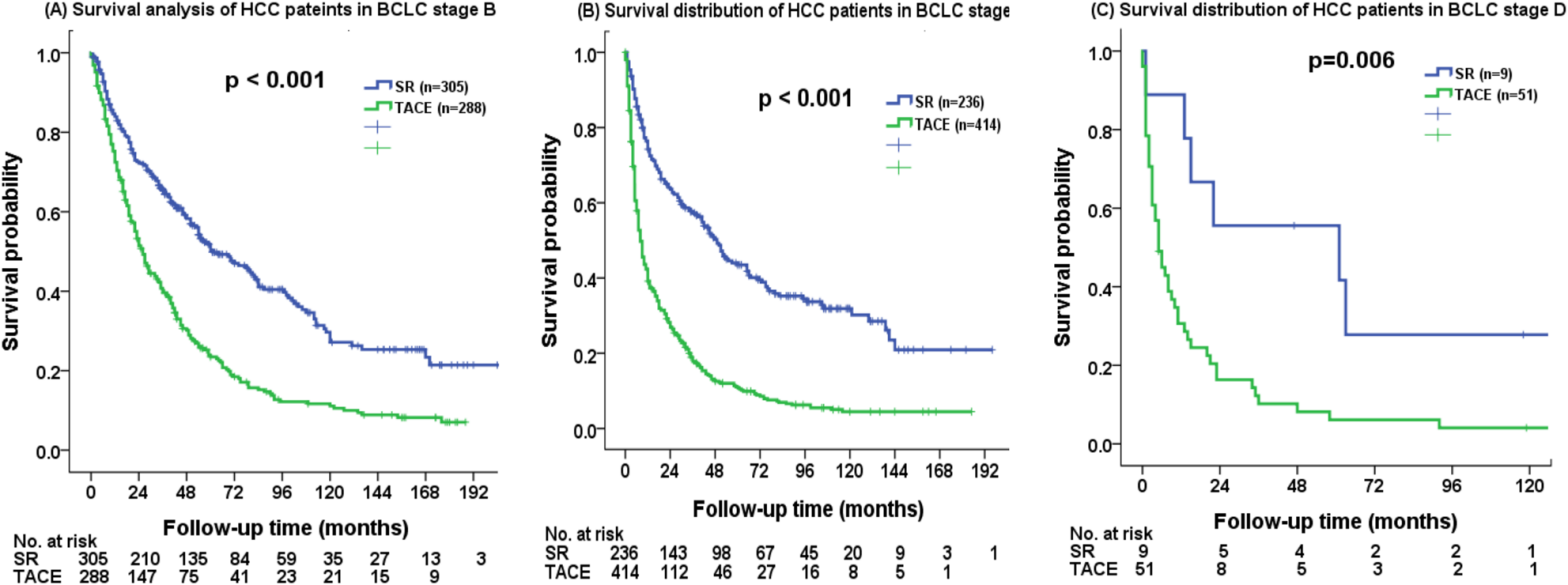
Comparison of overall survival in HCC patients beyond the Milan criteria undergoing surgical resection (SR) and transarterial chemoembolization (TACE) based on (**A**) BCLC stage B, (**B**) BCLC stage C, and (**C**) BCLC stage D. SR group consistently had a better survival compared with TACE group in patients with different BCLC stages (all *p* < 0.01).

The comparison of survival between patients undergoing SR and TACE based on BCLC stages was performed. The SR group consistently had a better overall survival in BCLC stage B (Fig. 4A, p < 0.001), stage C (Fig. 4B, p < 0.001) and stage D (Fig. 4C, p = 0.006) patients.

### Multivariate cox analysis

In univariate analysis of the entire cohort (Table 2), factors including older age, lower serum albumin level, higher bilirubin level, higher creatinine level, higher international normalized ratio of prothrombin time (INR of PT), AFP > 200 ng/mL, performance status 2–4, medium TBS, high TBS, ALBI grade 2–3, vascular invasion and TACE therapy, were significantly associated with decreased survival. Multivariate Cox analysis revealed that age > 66 years (HR: 1.145, 95% CI: 1.004–1.305, *p* = 0.043), AFP > 200 ng/mL (HR: 1.602, 95% CI: 1.402–1.831, *p* < 0.001), performance status 2–4 (HR: 1.316, 95% CI: 1.115–1.553, *p* < 0.001), medium TBS (HR: 1.225, 95% CI: 1.045–1.436, *p* = 0.012), high TBS (HR: 1.976, 95% CI: 1.637–2.384, *p* < 0.001), ALBI grade 2–3 (HR: 1.529, 95% CI: 1.342–1.743, *p* < 0.001), vascular invasion (HR: 1.568, 95% CI: 1.354–1.816, *p* < 0.001), and TACE (HR: 2.396, 95% CI: 2.082–2.759, *p* < 0.001) independently predicted a shortened survival.

**Table 2:** Multivariate survival analysis in HCC patients beyond Milan criteria undergoing surgical resection or transarterial chemoembolization (n = 1303).

Overall survival	Univariate analysis			Multivariate analysis		
	HR	CI	*p*	HR	CI	*p*
Age (≦66/ > 66 years)	1.242	1.096–1.470	< 0.001	1.145	1.004–1.305	0.043
Sex (male/female)	0.975	0.834–1.139	0.747			
HBsAg (negative/positive)	0.918	0.810–1.040	0.179			
Anti-HCV (negative/positive)	0.870	0.754–1.003	0.055			
Albumin level (≥ 3.5/ < 3.5 g/dL)	1.854	1.617–2.127	< 0.001			
Bilirubin level (≦1.1/ > 1.1 mg/dL)	1.379	1.201–1.582	< 0.001			
ALT (≦40/ > 40 IU/L)	0.917	0.808–1.041	0.181			
Creatinine (≦1.1/ > 1.1 mg/dL)	1.176	1.027–1.347	0.012			
Platelet (≥ 150,000/ < 150,000/μL)	0.912	0.802–1.038	0.162			
INR of PT (≦1.0/ > 1.0)	1.483	1.292–1.702	< 0.001			
AFP (≦200/ > 200 ng/mL)	1.693	1.491–1.921	< 0.001	1.602	1.402–1.831	< 0.001
Performance status 0–1/2–4	1.835	1.562–2.156	< 0.001	1.316	1.115–1.553	< 0.001
Tumor burden score						
Low	1			1		
Medium	1.215	1.040–1.419	< 0.001	1.225	1.045–1.436	0.012
High	2.225	1.861–2.660	< 0.001	1.976	1.637–2.384	< 0.001
ALBI grade 1/grade2–3	1.828	1.608–2.079	< 0.001	1.529	1.342–1.743	< 0.001
Vascular invasion (no/yes)	1.892	1.641–2.181	< 0.001	1.568	1.354–1.816	< 0.001
SR versus TACE	2.494	2.181–2.852	< 0.001	2.396	2.082–2.759	< 0.001

The forepart of the parentheses was set as the reference group in the univariate and multivariate analysis.

*AFP* ɑ-fetoprotein, *ALBI* albumin-bilirubin, *ALT* alanine aminotransferase, *INR of PT* international normalized ratio of prothrombin time, *SR* surgical resection, *TACE* transarterial chemoembolization, *TBS* tumor burden score.

In univariate analysis of patients with low TBS (n = 319, Table 3), older age, positive anti-HCV, lower serum albumin level, higher bilirubin level, higher creatinine level, thrombocytopenia, higher INR of PT, AFP > 200 ng/mL, performance status 2–4, ALBI grade 2–3, vascular invasion and TACE, were associated with decreased long-term survival. Multivariate analysis revealed that age > 66 years (HR: 1.517, 95% CI: 1.515–2.000, *p* = 0.003), AFP > 200 ng/mL (HR: 1.811, 95% CI: 1.345–2.439, *p* < 0.001), performance status 2–4 (HR: 1.888, 95% CI: 1.264–2.822, *p* < 0.001), ALBI grade 2–3 (HR: 1.738, 95% CI: 1.317–2.294, *p* < 0.001), vascular invasion (HR: 1.537, 95% CI: 1.102–2.142, *p* < 0.001), and TACE (HR: 1.683, 95% CI: 1.246–2.274, *p* < 0.001) were linked with increased mortality.

**Table 3:** Multivariate survival analysis of HCC patients with low TBS undergoing surgical resection or transarterial chemoembolization (n = 319).

Overall survival	Univariate analysis			Multivariate analysis		
	HR	CI	*p*	HR	CI	*p*
Age (≦66/ > 66 years)	1.794	1.381–2.330	< 0.001	1.517	1.151–2.000	0.003
Sex (male/female)	0.799	0.581–1.044	0.095			
HBsAg (negative/positive)	0.859	0.664–1.112	0.249			
Anti-HCV (negative/positive)	1.442	1.105–1.880	0.007			
Albumin level (≥ 3.5/ < 3.5 g/dL)	2.390	1.771–3.225	< 0.001			
Bilirubin level (≦1.1/ > 1.1 mg/dL)	1.330	1.014–1.746	0.040			
ALT (≦40/ > 40 IU/L)	0.944	0.727–1.226	0.664			
Creatinine (≦1.1/ > 1.1 mg/dL)	1.330	1.002–1.766	0.048			
Platelet (≥ 150,000/ < 150,000/μL)	1.528	1.172–1.992	0.002			
INR of PT (≦1.0/ > 1.0)	0.762	0.574–1.010	0.059			
AFP (≦200/ > 200 ng/mL)	1.450	1.086–1.937	0.012	1.811	1.345–2.439	< 0.001
Performance status 0–1/2–4	2.270	1.541–3.342	< 0.001	1.888	1.264–2.822	0.002
ALBI grade 1/grade2–3	1.949	1.494–2.543	< 0.001	1.738	1.317–2.294	< 0.001
Vascular invasion (no/yes)	1.892	1.641–2.181	< 0.001	1.537	1.102–2.142	0.011
SR versus TACE	2.134	1.615–2.820	< 0.001	1.683	1.246–2.274	< 0.001

The forepart of the parentheses was set as the reference group in the univariate and multivariate analysis.

*AFP* ɑ-fetoprotein, *ALBI* albumin-bilirubin, *ALT* alanine aminotransferase, *INR of PT* international normalized ratio of prothrombin time, *SR* surgical resection, *TACE* transarterial chemoembolization, *TBS* tumor burden score.

In univariate analysis of medium TBS group (n = 685, Table 4), older age, positive HBsAg and anti-HCV, lower albumin level, higher bilirubin level, higher creatinine level, thrombocytopenia, higher INR of PT, AFP > 200 ng/mL, performance status 2–4, ALBI grade 2–3, vascular invasion and TACE, were associated with an unfavorable outcome. Multivariate analysis revealed that AFP > 200 ng/mL (HR: 1.563, 95% CI: 1.302–1.877, *p* < 0.001), performance status 2–4 (HR: 1.293, 95% CI: 1.031–1.621, *p* < 0.001), ALBI grade 2–3 (HR: 1.598, 95% CI: 1.331–1.919, *p* < 0.001), vascular invasion (HR: 1.559, 95% CI: 1.260–1.930, *p* < 0.001), and TACE (HR: 2.575, 95% CI: 2.122–3.125, *p* < 0.001) were associated with decreased long-term survival.

**Table 4:** Multivariate survival analysis of HCC patients with medium TBS undergoing surgical resection or transarterial chemoembolization (n = 685).

Overall survival	Univariate analysis			Multivariate analysis		
	HR	CI	*p*	HR	CI	*p*
Age (≦66/ > 66 years)	1.279	1.072–1.526	0.006			
Sex (male/female)	0.976	0.783–1.216	0.826			
HBsAg (negative/positive)	1.208	1.014–1.441	0.035			
Anti-HCV (negative/positive)	1.265	1.032–1.550	0.024			
Albumin level (≥ 3.5/ < 3.5 g/dL)	1.815	1.501–2.195	< 0.001			
Bilirubin level (≦1.1/ > 1.1 mg/dL)	1.518	1.250–1.845	< 0.001			
ALT (≦40/ > 40 IU/L)	0.929	0.778–1.108	0.410			
Creatinine (≦1.1/ > 1.1 mg/dL)	0.944	0.780–1.143	0.557			
Platelet (≥ 150,000/ < 150,000/μL)	1.355	1.132–1.621	0.001			
INR of PT (≦1.0/ > 1.0)	1.533	1.257–1.871	< 0.001			
AFP (≦200/ > 200 ng/mL)	1.571	1.314–1.880	< 0.001	1.563	1.302–1.877	< 0.001
Performance status 0–1/2–4	1.731	1.386–2.161	< 0.001	1.293	1.031–1.621	0.026
ALBI grade 1/grade2–3	1.845	1.541–2.210	< 0.001	1.598	1.331–1.919	< 0.001
Vascular invasion (no/yes)	1.821	1.475–2.248	< 0.001	1.559	1.260–1.930	< 0.001
SR versus TACE	2.707	2.242–3.267	< 0.001	2.575	2.122–3.125	< 0.001

The forepart of the parentheses was set as the reference group in the univariate and multivariate analysis.

*AFP* ɑ-fetoprotein, *ALBI* albumin-bilirubin, *ALT* alanine aminotransferase, *INR of PT* international normalized ratio of prothrombin time, *SR* surgical resection, *TACE* transarterial chemoembolization, *TBS* tumor burden score.

In univariate analysis of high TBS group (n = 299, Table 5), older age, positive HBsAg and anti-HCV, lower albumin level, higher bilirubin level, higher creatinine level, thrombocytopenia, higher INR of PT, AFP > 200 ng/mL, performance status 2–4, ALBI grade 2–3, vascular invasion, and TACE were linked with a shortened survival. Multivariate analysis disclosed that AFP > 200 ng/mL (HR: 1.483, 95% CI: 1.150–1.913, *p* = 0.002), ALBI grade 2–3 (HR: 1.428, 95% CI: 1.100–1.855, *p* = 0.007), vascular invasion (HR: 1.499, 95% CI: 1.160–1.938, *p* = 0.002), and TACE (HR: 2.580, 95% CI: 1.964–3.390, *p* < 0.001) were independent predictors associated with decreased survival.

**Table 5:** Multivariate survival analysis in HCC patients with high TBS undergoing surgical resection or transarterial chemoembolization (n = 299).

Overall survival	Univariate analysis			Multivariate analysis		
	HR	CI	*p*	HR	CI	*p*
Age (≦66/ > 66 years)	0.975	0.758–1.255	0.845			
Sex (male/female)	0.891	0.631–1.258	0.512			
HBsAg (negative/positive)	0.998	0.774–1.285	0.249			
Anti-HCV (negative/positive)	0.968	0.693–1.353	0.850			
Albumin level (≥ 3.5/ < 3.5 g/dL)	1.567	1.201–2.044	0.001			
Bilirubin level (≦1.1/ > 1.1 mg/dL)	1.536	1.155–2.043	0.003			
ALT (≦40/ > 40 IU/L)	0.937	0.727–1.208	0.617			
Creatinine (≦1.1/ > 1.1 mg/dL)	1.330	1.018–1.738	0.036			
Platelet (≥ 150,000/ < 150,000/μL)	0.986	0.677–1.436	0.942			
INR of PT (≦1.0/ > 1.0)	1.557	1.197–2.025	0.001			
AFP (≦200/ > 200 ng/mL)	1.528	1.188–1.966	0.001	1.483	1.150–1.913	0.002
Performance status 0–1/2–4	1.638	1.212–2.215	< 0.001			
ALBI grade 1/grade2-3	1.570	1.215–2.030	0.001	1.428	1.100–1.855	0.007
Vascular invasion (no/yes)	1.722	1.340–2.214	< 0.001	1.499	1.160–1.938	0.002
SR vs TACE	2.826	2.161–3.695	< 0.001	2.580	1.964–3.390	< 0.001

The forepart of the parentheses was set as the reference group in the univariate and multivariate analysis.

*AFP* ɑ-fetoprotein, *ALBI* albumin-bilirubin, *ALT* alanine aminotransferase, *INR of PT* international normalized ratio of prothrombin time, *SR* surgical resection, *TACE* transarterial chemoembolization, *TBS* tumor burden score.

## Discussion

According to the Barcelona Clinic Liver Cancer (BCLC) staging system, TACE is suggested for multinodular HCC beyond the Milan criteria, whereas SR is mainly indicated in early stage HCC^5,20^. However, independent studies reported that SR could also be performed in multinodular HCC with adequate liver reserve^27–32^. In this study, we compared SR and TACE in HCC patients beyond the Milan criteria specifically based on a new biomarker, TBS. Our results show that SR may provide better survival compared with TACE in these patients. Notably, SR was associated with improved survival in both entire cohort and subgroup patients with different TBS distribution. Therefore, SR is superior to TACE in patients with advanced HCC, and the survival benefit is not influenced by TBS. In addition, TBS is confirmed as an independent prognostic predictor to discriminate long-term outcome in this special patient group.

The prognosis of HCC beyond the Milan criteria is highly variable due to their heterogeneous tumor burden and liver functional reserve. Traditionally, the binary fashion of the diameter of tumor and number of nodules represent the extent of tumor burden in HCC, and these variables are included in the BCLC staging system. Other models, such as up-to-seven criteria, up-to-eleven criteria and seven-eleven criteria, use categorical cutoffs which could weaken the prognostic power due to inaccurate causal inferences. Thus, the paradigm shift from dichotomous to continuous measurement of tumor burden would increase prognostic stratification for cancer patients. Recently, TBS was proposed to indicate tumor burden in HCC, and has been validated by several independent studies^16,17,33^. There are apparently several advantages of using TBS to represent tumor burden. First, TBS is a simple and continuous measurement of the extent of tumor involvement based solely on the largest tumor diameter and number of nodules. Second, TBS can be conveniently classified into different risk groups to estimate the outcome more specifically. Third, there is a clear dose–response relationship between TBS and patient outcome. Our data show that patients with medium and high TBS were associated with increased risk of mortality compared with those with low TBS in the multivariate analysis. These results are consistent with previous studies^18,33,34^, and support the notion that TBS is a feasible prognostic surrogate to predict long-term outcome in HCC patients beyond the Milan criteria.

Treatment modality is a crucial determinant to predict long-term survival in HCC patients. TACE is recommended for intermediate stage HCC, but the role of SR for this patient stage is quite controversial based on current HCC practice guidelines^5,6^. Due to continuous improvement in surgical technique and perioperative care, SR is also suggested for selected patients with multinodular HCCs. Our findings show that SR group had better long-term survival compared with the TACE group. Notably, patients undergoing TACE had 2.4-fold increased risk of mortality compared with SR group in multivariate analysis. In subgroup analysis, we confirm that SR may provide better long-term survival compared with TACE in different TBS distribution. We also demonstrate that surgical HCC patients beyond the Milan criteria may achieve 5-year survival rate of 49% which is comparable with a previous multi-institutional international study^33^. Our findings are also in accordance with most previous studies^10,27–29,35,36^, and confirm that SR is a favorable prognostic factor for long-term outcome in advanced stage HCC independent of the grade of TBS.

The severity of liver dysfunction is known to play a critical role in treating HCC. In our study, patients with ALBI grade 2–3 had 1.5-fold increased risk of mortality compared with those of ALBI grade 1 in the multivariate analysis. Notably, in subgroup analysis, ALBI grade 1 patients consistently had better long-term outcome than ALBI grade 2–3 patients in different TBS distributions. The results are in line with previous studies^23–25^, and support the clinical feasibility of TBS for outcome prediction. Performance status is also linked with the prognosis of HCC. Our findings unequivocally show that patients with poor performance status were associated with decreased survival. Moreover, other predictors, including vascular invasion and high AFP level, were also associated with poor prognosis in HCC as demonstrated in our and previous studies^26,37^. Taken altogether, the extent of tumor burden, liver functional reserve, performance status and tumor behavior are independent prognostic predictors in HCC beyond the Milan criteria.

This study has a few potential limitations. Firstly, this is a single center study in Asia–Pacific region where hepatitis B is the predominant etiology of HCC that is quite different from most western countries and Japan. Secondly, TBS is a simple and continuous variable to estimate tumor burden, but the diameter and number of nodules represent the same statistical weight that could introduce bias in calculation. Thirdly, treatment selection for HCC was primarily based on the decision from physicians and patients, and may not completely comply with current BCLC recommendations.

In conclusion, TBS is an independent prognostic predictor in HCC beyond the Milan criteria. SR consistently provides better long-term outcome compared with TACE in these patients independent of TBS. SR should be considered as the primary treatment modality in selected patients with advanced stage HCC. Further study is required to validate our findings.

## Data Availability

The datasets generated and/or analysed during the current study are not publicly available due to ethical reasons but are available from the corresponding author on reasonable request.
